# Infectious Disease Prevalence and Factors Associated with Upper Respiratory Infection in Cats Following Relocation

**DOI:** 10.3390/ani8060091

**Published:** 2018-06-09

**Authors:** Mehnaz Aziz, Stephanie Janeczko, Maya Gupta

**Affiliations:** 1 Shelter Outreach, American Society for the Prevention of Cruelty to Animals (ASPCA^®^), New York, NY 10018, USA; stephanie.janeczko@aspca.org; 2 Strategy, Research and Development, American Society for the Prevention of Cruelty to Animals (ASPCA^®^), New York, NY 10018, USA; maya.gupta@aspca.org

**Keywords:** animal relocation, transport, animal shelter, cat, infectious disease, transfer, animal welfare, feline upper respiratory infection, feline panleukopenia virus, dermatophytosis

## Abstract

**Simple Summary:**

Relocation of cats and kittens is a relatively new practice in animal welfare. It is one of the many tools used by animal welfare agencies to decrease shelter euthanasia rates across the country. However, there are few and sometimes conflicting guidelines for either minimum standards or best practices regarding relocation programs. Most operational practices are evolving and are often based on lessons learned. Concerns about the frequency of infectious diseases and the corresponding likelihood of spread are commonly raised in the context of animal relocation. In this study, which followed one relocation program over a 7-month period, highly contagious infectious diseases, feline panleukopenia virus (FPV) and ringworm, were uncommon in cats following relocation into one shelter. Upper respiratory infection (URI) was, however, relatively more frequent with younger age, increased time in transport during relocation and increased time spent at the shelter following relocation all associated with increased disease frequency. Accordingly, even in an established relocation program, steps should be taken to mitigate the risk of upper respiratory infection in relocated cats.

**Abstract:**

Feline relocation is used increasingly in animal welfare to decrease shelter euthanasia rates and increase positive outcomes. Concerns about infectious disease introduction and transmission are often expressed; however, little research has been conducted on even the baseline prevalence of infectious disease following relocation. This study, which collected data on 430 cats relocated through an established program over 7 months, evaluated the prevalence of upper respiratory infection (URI), feline panleukopenia virus (FPV) and dermatophytosis at one destination agency. The period prevalence was 25.8% for URI, 1.6% for FPV and 0.9% for dermatophytosis. Mixed-effects logistic regression was performed to investigate factors associated with URI. Younger age, increased time in transport, and increased length of stay at the destination agency were associated with increased URI prevalence following relocation. The findings of this study reveal that certain highly contagious and environmentally persistent infectious diseases, such as FPV and dermatophytosis, are uncommon following relocation in an established program; however, URI in relocated cats should be proactively managed. Animal welfare agencies can use this information to guide shelter and relocation operations and mitigate the impact of URI in relocated cats.

## 1. Introduction

According to the American Society for the Prevention of Cruelty to Animals (ASPCA), 6.5 million animals enter shelters in the United States annually [1]. Cats account for 3.2 million of these animals, approximately 26.9% of which are euthanized [1]. In an effort to decrease shelter euthanasia rates and expand opportunities for positive outcomes, companion animal relocation programs have been developed across the country. These programs typically relocate animals from source agencies with limited resources to destination agencies with greater capacity to support the needs of animals and promote positive outcomes.

A combination of federal, state and/or municipal regulations for transporting animals are in place, but these regulations vary on the state and local levels. Additionally, they do not necessarily specify how best to uphold animal welfare and well-being, public safety and disease prevention in relocation programs. Accordingly, resources on best practices for companion animal relocation have been put forth in the Association of Shelter Veterinarians’ *Guidelines for Standards of Care in Animal Shelters*, the American Veterinary Medical Association’s *Best Practices for the Relocation of Dogs and Cats for Adoption* and the Society of Animal Welfare Administrators’ *Companion Animal Transport Programs Best Practices* [2,3,4]. However, as a relatively new and growing practice, operational procedures and best practices for relocating cats are evolving and conflicting recommendations sometimes exist. One study examining canine relocation indicated increased transmission of vector-borne diseases following disaster response [5], and significant concerns about the potential for disease transmission when making transfer-related decisions have been reported by animal welfare personnel [6]. In addition, state veterinarians and organized veterinary medicine have expressed similar concerns surrounding relocation and infectious disease transmission [7,8]. The risk of unintentional introduction and transmission of feline infectious diseases through relocation, however, is currently based on anecdotal experience [9].

The transport of apparently healthy animals that may be subclinically harboring pathogens, the temporary housing of multiple animals in close quarters within a transport vehicle and the stress of transport itself may lead to increased susceptibility to infection and elevated risk of disease transmission [2,4]. Feline upper respiratory infection (URI) in shelters, primarily driven by feline herpesvirus, has specifically been linked to stress and is a concern surrounding relocation [10,11,12]. Additional concerns associated with other infectious diseases, such as feline panleukopenia (FPV) and dermatophytosis, include the severity of disease, the environmental stability of the pathogens and the potential for zoonotic transmission (dermatophytosis only). While these infectious diseases can negatively impact destination agencies or the communities they serve, limited data exists to quantify the level of risk [9]. Understanding the prevalence of common infectious diseases in relocated cats will provide data regarding frequency of such conditions and could help inform future recommendations regarding measures to limit unintentional disease introduction and transmission. 

The objective of this study was to determine the prevalence of URI, FPV and dermatophytosis in cats transported through an established relocation program in the state of Washington. A secondary objective was to investigate factors associated with these infectious diseases. 

## 2. Materials and Methods 

### 2.1. Study Site and Population

This study was performed at a privately operated animal shelter in Washington from May 2016 to November 2016. Shelter admission consisted of dogs, cats and small mammals. Animals were admitted from the public as well as through the shelter’s relocation program from a variety of source agencies both inside and outside of the community. Total cat intake for the shelter in 2016 was 1461, with a cat adoption rate of 91%. 

Study data were collected from all 430 cats admitted to the shelter via the relocation program during the study period in 2016. Relocated cats of all ages, sexes and breeds were eligible for inclusion in the study. Per the shelter’s general relocation program protocol, cats that were transported in had to appear healthy via visual screening by trained shelter staff prior to and on the day of relocation and had to have received a modified live feline viral rhinotracheitis, calicivirus and panleukopenia vaccine (FVRCP) prior to relocation if they were 4 weeks of age or older. Cats originating from outside of the state required a certificate of veterinary inspection (health certificate). The majority of cases followed these relocation criteria, with the exception of 9 individual kittens that were not FVRCP vaccinated prior to relocation despite being 4 weeks of age or older. 

### 2.2. Data Collection

Shelter staff observed each study cat daily at the destination agency for the development of URI, FPV and/or dermatophytosis based on the study’s case definitions (Table 1). The first date of development of illness consistent with the case definition was recorded for each cat if pertinent. Additional information was collected for each cat including age, source agency (originating agency), time in transport (hours spent in travel), outcome at destination (adopted, died or euthanized) and length of stay at destination (total days spent within destination agency). Characteristic information and disease information for the general population of cats at the destination agency (cats that were not transported in) were not collected.

### 2.3. Data Management and Statistical Analysis

All statistical analyses were performed using Stata (version 15, StataCorp, College Station, TX, USA). Descriptive analyses were performed on the data. The period prevalence rates of URI, FPV and dermatophytosis were calculated and reported as the number of relocated cats meeting the case definition divided by the total number of relocated cats.

The low rates of FPV and dermatophytosis in the sample precluded modeling for factors associated with those diseases. A mixed-effects logistic regression model was used to examine factors associated with URI while accounting for potential similarities among cats from the same source agency, including the likelihood of similar disease outcomes. Three variables were initially selected for inclusion in the model based on potential clinical importance: cat age, time in transport and length of stay at the destination agency. As described in Hosmer, Lemeshow and Sturdivant, initial screening based on univariable logistic regressions of URI on each variable revealed all three as candidates for inclusion in the full model using a selection cutoff, here set at *p* ≤ 0.1 [14]. Multilevel mixed-effects models include both random effects, which account for grouping of participants who may share similarities (in this case, cats from the same source agency) and traditional (fixed) effects. Accordingly, in the mixed-effects model, source agency was fitted with random intercepts using an independent covariance structure to model shared variance at the source agency level, while cat age, time in transport and length of stay at the destination agency were fitted as fixed effects. Model assumptions of (1) a linear relationship between the explanatory variables and the log odds of the outcome variable and (2) absence of substantial multicollinearity among the explanatory variables were tested in Stata using the boxtid and collin commands, respectively. Model goodness-of-fit was assessed using the global Wald statistic and the likelihood ratio test of the multilevel model versus an ordinary logistic regression. All analyses were performed using listwise deletion (cases with any missing observations were withheld from analysis), resulting in a total of 398 cat records analyzed for the mixed-effects model.

## 3. Results

### 3.1. Descriptive Data

From May to November 2016, 430 relocated cats entered the destination agency from 18 source agencies. The average age of cats was 1.6 years (range: 1 week to 15 years). There were 199 kittens (46.3%), defined as younger than five months of age, and 231 adult cats (53.7%) five months of age or older. The average time in transport was 17.6 h (range: 0.5 to 29 h).

Of the 398 relocated cats with known outcomes, the average length of stay at the destination agency was 18.1 days (range: 0 to 140 days). One hundred and sixty-seven cats (42.0%) remained in the destination agency for 7 days or less, 69 (17.3%) had an 8–14-day length of stay, 51 (12.8%) had a 15–21-day length of stay and 111 (27.9%) stayed for 22 days or more. Three hundred and seventy-nine cats (95.2%) were adopted, 9 (2.3%) died and 10 (2.5%) were euthanized. Twenty-two of the adopted cats (5.8%) were returned to the shelter within 30 days.

### 3.2. Infectious Diseases of Cats at the Destination Agency

Within the study population, 111 cases of URI, 7 cases of FPV and 4 cases of dermatophytosis were diagnosed during the study period at the destination agency. The period prevalence of each disease was 25.8%, 1.6% and 0.9%, respectively. None of the cats diagnosed with FPV were diagnosed with URI or dermatophytosis. Two of the cats diagnosed with dermatophytosis were also diagnosed with URI.

Of the 111 cats diagnosed with URI at the destination agency, the average age of affected cats was 1.2 years (range: 2 weeks to 8 years). Fifty-nine affected cats (53.2%) were kittens and 52 (46.9%) were adults. Affected cats came from 13 different source agencies. Average transport time of affected cats was 19.5 h (range: 0.5 to 29 h). Time to URI diagnosis after intake at the destination agency averaged 8.4 days (range: 0 to 52 days). Of the 91 cats diagnosed with URI who had known outcomes, average length of stay at the destination agency was 31.6 days (range: 3 to 140 days). Eighty-nine were adopted (97.8%), 1 died (1.1%) and 1 was euthanized (1.1%).

Six of the 7 cats diagnosed with FPV at the destination agency came from the same source agency on the same transport and were in transport for four hours, arriving at the destination agency on a single day. These 6 were kittens under 6 weeks of age (average age was 4.8 weeks). Their littermate status was unknown. All were diagnosed with FPV the day after arrival. The seventh cat was three years of age and was transported for 1.5 h. No date of diagnosis was available. Of the seven cats diagnosed with FPV, four were adopted and three died. The average length of stay of the four adopted cats was 48.5 days. The remaining three cats died six, seven and 20 days after intake into the destination agency, respectively.

Of the four cats diagnosed with dermatophytosis, one was a 20-week-old kitten and three were adults, ranging from one to three years of age. The affected cats came from three different source agencies. The 20-week-old kitten was in transport for 1.5 h and the three adult cats were in transport for 23, 23 and 29 h, respectively. Only one cat had an available date of diagnosis, which was 14 days post-intake at the destination agency. All four cats diagnosed with dermatophytosis were adopted from the destination agency, with an average length of stay of 36 days.

### 3.3. Statistical Analysis

Table 2 displays the results of the univariable logistic regressions used to screen variables for inclusion in the mixed-effects logistic regression model.

Table 3 displays the results of the mixed-effects logistic regression model. The overall model was significant, Wald χ^2^(5) = 57.6, *p* < 0.01. However, the mixed-effects logistic model represented no significant improvement in fit over a regular logistic model, χ^2^(1) = 1.9, *p* = 0.09. Because Stata’s boxtid command is not supported within mixed-effects regression, the regular logistic model was used to assess the assumption of a linear relationship between the explanatory variables and the log odds of URI. None of the variables departed significantly from linearity, upholding this assumption of the model. The mean variance inflation factor (VIF) was 1.1 and tolerance was between 0.9 and 1.0 for all explanatory variables, upholding the assumption of no substantial multicollinearity.

Age, time in transport and length of stay at the destination agency were significant factors (*p* < 0.05) associated with URI in this model (Table 3). Younger age, longer time in transport and longer length of stay were associated with increased URI prevalence at the destination agency.

## 4. Discussion

This is the first study to report the prevalence of infectious diseases in cats following transport through an established relocation program. In this study, URI was the most common feline infectious disease of those tracked at the destination agency after relocation. Although this study may not be representative of all relocation programs during all times of the year, the results indicate that significant concern regarding the introduction or transmission of FPV and dermatophytosis following transport may not be indicated.

While data on the prevalence of URI, FPV and dermatophytosis within shelter populations is limited in the literature, previous studies do provide some comparative data. The prevalence of clinical URI in American shelters has been documented in a handful of studies. One study, conducted in a large urban shelter in the northeastern United States, relayed an approximately 30% clinical URI prevalence [15]. Another study reported a 55% clinical URI prevalence across eight California shelters [16]. Yet another multi-shelter study, which investigated the risk of URI in adult cats in nine American shelters, documented an overall average annual URI rate of 17% but discovered a wide variation in the URI rate across the nine shelters ranging from 3% to over 29% [17]. Shelter URI prevalence varies widely due to different environmental, management and population-based factors, including feline housing, population density, sanitation practices and vaccination protocols [15,17]; however, the 25.8% prevalence rate for URI in this study was comparable to previously documented URI rates.

Although the baseline URI prevalence at the destination agency was unavailable for comparison, the URI prevalence in relocated cats, which was comparable to or slightly lower than those reported in other shelters across the United States, is interesting in light of how stressful relocation likely is for animals, particularly cats. Indeed, one study documented that 45% of cats start re-shedding feline herpesvirus after incurring a stressful event such as entering a new shelter or being rehoused [18]. The stress associated with hours of transport and being placed into a new shelter is likely to reactivate herpesvirus and contribute to clinical URI after a lag phase of 4–11 days [10]. Accordingly, a higher prevalence of URI in relocated cats should not be unexpected and in the authors’ opinion it is surprising that the URI prevalence documented in this study was not higher. It should also be noted that diagnostic testing was not required as part of the case definition for URI; as such, the degree of viral shedding of individual cats was unknown and the true prevalence of URI infection may have been underestimated. As it has been documented that herpesvirus shedding significantly increases within the first 7 days of shelter entry, it is likely that many cats were shedding virus in this study, whether clinically ill or not [16,19]. Although the cats in this study spent an undisclosed amount of time in previous shelters (their respective source agencies) and even though a direct comparison cannot be made to studies that diagnostically tested for disease, it is likely that the frequency of viral shedding was higher than the noted frequency of clinical cats. Accordingly, understanding that relocated animals are subjected to additional stress and potentially additional disease while in transport vehicles, as well as the fact that they may be subclinically shedding virus, the management of relocated cats should be similar to that of other shelter cats based on currently recognized best practices. Adequate and appropriate low-stress care, housing, sanitation, biosecurity measures and population density should be maintained to minimize the risk of URI development in all cats.

The prevalence of FPV within shelter populations is undocumented; therefore, comparing the 1.6% prevalence of FPV in this study to other shelters is not possible. Feline panleukopenia virus is enzootic in all parts of the United States but is more prevalent in high density populations such as catteries and shelters [20]. Accordingly, sporadic FPV cases and outbreaks are still seen throughout American shelters. It is important to note that six of the seven FPV cases were kittens from the same source agency, were transported in on the same day via a four hour trip and were all diagnosed with FPV one day after intake into the destination agency. It is unknown whether these kittens were littermates. Based on the disease course of FPV and date of FPV diagnosis, these kittens were likely already incubating FPV upon arrival to the destination agency although they were clinically healthy on the day of transport. Thorough screening of animals through physical examination prior to transport to ensure that animals are not showing even mild signs of illness is vital to limiting disease transmission related to relocation. Guidelines on animal relocation emphasize this best practice and state that all animals being transported should receive a medical examination by a trained animal care professional or veterinarian within 24 h of transport [2,3,4]. Ideally, screening animals for obvious signs of illness should be performed immediately prior to loading for transport, as well. The routine screening of healthy appearing animals through diagnostic testing means however is not recommended. Testing is costly and, for FPV specifically, cage-side diagnostic kits have a low positive predictive value when testing healthy animals, resulting in the possibility of an increased rate of false positive results. While diagnostic testing can be valuable if used appropriately, establishing clear and strong biosecurity, sanitation and disease recognition practices as well as increasing herd immunity through timely administration of core vaccines are the first lines of defense in mitigating disease transmission within shelters.

Reports on the prevalence of dermatophytosis within shelter populations are sparse. One study that sampled 200 shelter cats from four different geographical regions along the Pacific coast of the United States documented an overall prevalence of 5.5% [21]. The lower prevalence of dermatophytosis (0.9%) noted in the current study may be explained by geographical differences in sampling or may be related to the disease course itself. The 1–3 week incubation period of dermatophytosis may have resulted in some cats being diagnosed with dermatophytosis after adoption from the destination agency [22]. The date of diagnosis was unavailable for all but one dermatophytosis case, which was two weeks post-intake at the destination agency. It is difficult to differentiate whether this cat was already incubating dermatophytosis during relocation or if it acquired dermatophytosis at the destination agency. However, the likelihood of being exposed and acquiring dermatophytosis at the destination agency is low, based on the fact that there were no reports of other dermatophytosis cases in the cats at the destination agency during the study period.

Younger (five months or less) cats were more likely to be diagnosed with URI (based on the aforementioned case definitions) at the destination agency than were older cats within the study population. Previous studies have similarly found young age to be a predictor of URI in catteries and shelters [15,16,23]. Kittens are more susceptible to disease as maternal immunity wanes, and they suffer more severe clinical signs than adult cats [15,19]. More than half (53.2%) of the 111 cats affected with URI in this study were kittens younger than five months of age, and their average time to URI diagnosis was six days (range: 0 to 22 days). The incubation period for herpesvirus is 2–6 days for a newly exposed animal [10]. Accordingly, URI in kittens, who are perhaps less likely to be latently infected with herpesvirus compared to older cats, is possibly reflective of infections acquired as a result of novel exposure in the shelter. Although it is unknown whether foster care programs or other offsite placement options would reduce URI prevalence in relocated kittens, these study findings reinforce the value in adhering to established best practices in shelter medicine. This includes separating younger kittens, particularly those too young for adoption, from the general shelter population through appropriately segregated housing and/or placement outside of the shelter (i.e., foster care placement) to minimize the likelihood of exposure [24].

The development of URI was associated with increased time in transport. The potential for both increased stress and increased disease exposure associated with the physical act of relocation itself likely contributes to increased URI risk. The American Veterinary Medical Association’s *Best Practices for the Relocation of Dogs and Cats for Adoption* recommends that the maximum transport time to an intermediate or final destination should not be greater than 12 h [2]. Accordingly, the average time in transport in this study (17.6 h) is not recommended, and it is possible that other relocation programs, with otherwise similar operations but shorter transport times, may have lower rates of URI. Additional information regarding transport itself that could provide more insights on the environmental stress associated with transport include the method of transportation (e.g., air versus ground), the density of animals on transport, whether transports are multispecies, the ambient temperature within the transport vehicle, and/or whether animals from multiple source agencies are in the same transport vehicle.

Increased length of stay (over seven days at the destination agency) was associated with increased URI prevalence. Various studies have documented this as a risk factor for URI in shelter populations [15,16,25]. Increased length of stay increases chances of exposure to respiratory pathogens and increases the likelihood of viral reactivation of chronic or previous infection related to environmental stress in the shelter [16,19]. As explained in Dinnage et al., requiring longer lengths of stay at shelters, such as intake quarantine periods, increases the URI incidence in shelters due to the pathophysiology and clinical nature of URI in shelter populations [15]. This study’s findings, while not establishing causality, are consistent with the interpretation that increased time spent in the shelter is associated with increased URI prevalence. Accordingly, minimizing shelter length of stay is critical for URI management from an individual animal perspective as a key method of limiting disease exposure and environmental stress. From a population-based perspective, minimizing shelter length of stay is equally critical in that it maximizes shelter resources, capacity and population flow while simultaneously taking animal well-being into account. Regardless of whether an animal acquires URI due to increased length of stay or whether an animal with URI has a longer stay in the destination agency, increased length of stay related to URI is undesirable in any shelter and has negative impacts on the entire population.

Length of stay may also be impacted by state or municipal mandates that require intake quarantine periods of relocated animals, as may the relocation of animals from source communities with known high prevalence rates of FPV or dermatophytosis. However, the low number of FPV and ringworm cases noted in this study suggest that quarantine periods for relocated cats should be carefully considered as they may contribute to development of other diseases such as URI without meaningfully reducing risk [15]. While applicable state and municipal regulations surrounding quarantine of relocated animals must be followed, shelters should carefully consider the risks and benefits of voluntarily imposed intake quarantine periods. The risk of infectious disease should be balanced with the shelter’s capacity and resources related to quarantine. Animal health status, source and any known infectious disease risk within the source, as well as incubation periods of pathogens of concern should be weighed against increased costs, reduced shelter capacity and the potential adverse health effects of increasing length of stay in the shelter [4]. Routine disease surveillance and the prompt identification and isolation of clinically ill animals can instead be used to mitigate infectious disease concerns [15,26].

To the authors’ knowledge, this is the first study on infectious disease prevalence surrounding feline relocation to provide data-based insights on otherwise anecdotal concerns regarding animal relocation. However, the study design did have limitations. First, no information was available on feline health status, environmental factors or average length of stay of cats at the source agencies. This study focused only on the population of cats that were transported and all cats had to be overtly healthy on the day of transport to be eligible for relocation. Accordingly, this study did not investigate the general population of shelter cats that would have initially been eligible and/or selected for relocation but may have been excluded from relocation as a result of the subsequent development of physical and/or behavioral health concerns within source agencies. For example, investigation of the total length of stay of cats within the source and destination agencies is warranted, since this study and previous studies have shown that increased length of stay is a risk factor for URI development [15,16,25]. The time to onset of URI, whether driven by novel exposure to a respiratory pathogen or the recrudescence of latent feline herpesvirus, is important to consider in light of length of stay at both the source and destination agencies and can convey both a causal and temporal relationship between URI and length of stay. Future investigations could prospectively follow cats in a case-control study within source agencies to their outcomes to create a more accurate and comprehensive representation of the infectious disease prevalence and risks surrounding relocation.

Second, information regarding other cats in the destination agency, specifically those that were not transported in, was unavailable. In addition, information regarding baseline feline infectious disease rates of the destination agency was unavailable. Accordingly, an infectious disease incidence rate at the destination agency, which would have incorporated how many cats were at-risk of developing disease on a daily basis throughout the facility, was not calculated. A disease incidence rate would have provided a contrasting view of the infectious disease risk for relocated cats versus other cats in the destination agency, thereby providing an a more comprehensive understanding of which feline populations are most at-risk of infectious diseases.

While this study focused on factors associated with URI at the destination agency, it did not investigate all potential factors that might contribute to shelter URI within the destination agency, such as sanitation and biosecurity practices, population density or housing. The factors assessed in this study may have been associated with other such factors and/or have been associated with the other explanatory factors in the model themselves. In addition, this was a preliminary study that observed disease at the destination agency and did not focus on the development of disease following adoption. Challenges in obtaining and confirming dates and diagnoses for conditions reported by adopters precluded the usefulness of such post-adoption data. It should also be noted that the case definition-based method of URI diagnosis in this study may have resulted in overestimation or underestimation of disease. Lastly, the study was a convenience sample of cats in one geographic location over a period of seven months and may not reflect the infectious disease prevalence and risk factors associated with the general shelter cat population across the United States during all times of the year. Because seasonal variations can affect infectious disease prevalence, this study was purposely conducted over the summer season to ensure a more conservative estimate of infectious disease prevalence was reflected as this is when shelters are typically faced with higher intake numbers and population density related to the increased birth rate of cats. Future studies are needed to continue to explore how relocation programs impact feline health on both the source and destination sides.

## 5. Conclusions

Relocation programs allow for the transfer of animals from source agencies with limited resources to destination agencies with a greater capacity to support and provide positive outcomes for animals. This study reveals that highly contagious feline infectious diseases that can result in high mortality and morbidity of cats, such as FPV and dermatophytosis, were uncommon following transport in one established relocation program. The development of URI in shelters, which has been closely associated with stress, is a predictable concern when relocating cats considering that transport itself, as well as the introduction to a new environment, are stressful events. Factors associated with URI in cats following transport include younger age, longer time in transport and increased length of stay at the destination agency. The prevalence of infectious disease noted in this study is based on the transport of overtly healthy animals in an established relocation program. Although these data are collected from one relocation period over one period of time, they can be used to improve the understanding of factors associated with disease and provide direction and focus for future research. Importantly, this information can help shelters develop protocols around relocated animals to reduce disease transmission within the shelter. Documented strategies that can minimize the risk of developing URI in shelters should be followed for relocated cats at destination agencies, including the provision of larger housing units, minimization of movement of cats within and between cages, utilization of appropriate sanitation methods, minimization of length of stay within the shelter and reduction of shelter population density [17,27].

## Figures and Tables

**Table 1:** Case definitions for URI, FPV and dermatophytosis.

**Upper Respiratory Infection**	One or more of the following clinical signs: ocular discharge, conjunctivitis, chemosis, nasal discharge, sneezing or oral ulceration.
	Laboratory testing was not required.
**Feline Panleukopenia Virus**	One or more of the following clinical signs: sudden death, vomiting, diarrhea, lethargy or inappetence AND
	One or more of the following test results: positive fecal canine parvovirus enzyme-linked immunosorbent assay (ELISA), positive feline panleukopenia virus polymerase chain reaction (PCR), or blood smear qualitatively consistent with leukopenia or complete blood count (CBC) of less than 3000 white blood cells/μL (normal range: 5500–19,500 white blood cells/μL) [13].
**Dermatophytosis**	One or more of the following clinical signs commonly associated with dermatophytosis: hair loss, easily broken hairs, or scaling and/or redness of the skin, particularly when noted on the face/head, toes or medial aspects of the front legs AND/OR
	A positive Wood’s lamp examination or fungal culture result.

**Table 2:** Univariable logistic regressions of factors associated with URI at the destination agency.

Variable	Category	OR (95% CI)	*p*-Value
Age (*n* = 430)	<5 months	Referent	---
	≥5 months	0.7 (0.5–1.1)	0.1
Time in transport (*n* = 430)	N/A	1.0 (1.0–1.1)	0.0 *
Length of stay at destination agency (*n* = 398)	≤7 days	Referent	---
	8–14 days	3.3 (1.4–7.8)	0.0 *
	15–21 days	6.5 (2.8–15.2)	0.0 *
	≥22 days	12.1 (5.9–24.7)	0.0 *

* p < 0.05.

**Table 3:** Fixed and random effects logistic regression estimates of factors associated with URI at the destination agency (*n* = 398).

Variable	Category	OR (95% CI)	*p*-Value
		**Fixed Effects**	
Age	<5 months	Referent	---
	≥5 months	0.3 (0.2–0.6)	0.0 *
Time in transport	N/A	1.1 (1.0–1.1)	0.0 *
Length of stay at destination agency	≤7 days	Referent	---
	8–14 days	3.8 (1.5–9.3)	0.0 *
	15–21 days	9.8 (3.9–24.8)	0.0 *
	≥22 days	20.9 (9.2–47.3)	0.0 *
		**Random effects**	
Source agency	N/A	0.2 (0.0–2.5)	---

* *p* < 0.05.
